# Relationship between vitamin D serum concentration and inflammatory bowel disease demographics: A cross-sectional study

**DOI:** 10.1097/MD.0000000000051257

**Published:** 2026-10-16

**Authors:** Mohammad Shehab, Abdulwahab AlSayegh, Ali H. Ziyab

**Affiliations:** a Program of Medicine, College of Medicine and Health Sciences, Abdullah Al Salem University, Khaldiya, Kuwait; b Division of Gastroenterology, Department of Internal Medicine, Mubarak AlKabeer Hospital, Jabriya, Kuwait; c Department of Community Medicine and Behavioral Sciences, College of Medicine, Kuwait University, Kuwait City, Kuwait.

**Keywords:** Crohn disease, inflammatory bowel disease, ulcerative colitis, Vitamin D, Vitamin D deficiency

## Abstract

Vitamin D deficiency is increasingly recognized as a common comorbidity in patients with inflammatory bowel disease (IBD), potentially influencing immune regulation and disease outcomes. Despite high rates of vitamin D deficiency reported globally, data from the Middle East remain scarce. This study aimed to evaluate the relationship between vitamin D concentration and the demographics of patients with IBD. We conducted a cross-sectional comparative study utilizing an IBD clinical cohort from a tertiary care center and a separate, previously published population-based control cohort. Serum 25-hydroxyvitamin D [25(OH)D] concentrations were measured in 184 patients with IBD (122 Crohn disease, 62 ulcerative colitis) and 410 controls without IBD. Associations between vitamin D concentrations, demographics, and disease subtype were assessed using chi-squared tests, Wilcoxon rank-sum tests, and logistic regression to calculate adjusted odds ratios (aOR). IBD patients had significantly lower median vitamin D concentrations than controls (18.0 vs 31.0 nmol/L, *P* < .001), with 78.8% exhibiting severe deficiency (<25 nmol/L). Severe vitamin D deficiency was associated with higher odds of IBD in both sexes, with a stronger association among females (aOR 10.57, 95% CI: 5.33–20.96) than males (aOR 4.64, 95% CI: 2.57–8.37; *P*-interaction = .021). Vitamin D concentrations were significantly lower in Crohn disease compared to ulcerative colitis (16.5 vs 22.0 nmol/L, *P* = .003), though no associations were found with CD phenotype, and UC extent showed no association overall. Vitamin D deficiency is highly prevalent among patients with inflammatory bowel disease, particularly in those with Crohn disease and in females, underscoring both systemic disease-related associations and sex-specific vulnerability. Larger, prospective, multicenter studies are warranted to further elucidate the associated factors and clinical implications of vitamin D deficiency in this population.

## 1. Introduction

Inflammatory bowel diseases (IBD) are chronic, relapsing inflammatory disorders of the gastrointestinal tract, encompassing Crohn disease (CD) and ulcerative colitis (UC). While CD can involve any part of the gastrointestinal tract, UC is typically confined to the colon and rectum. Although their pathogenesis is not fully understood, IBD is recognized as a multifactorial condition involving genetic susceptibility, environmental exposures, gut microbiota, and immune dysregulation.

The global burden of IBD has been rising steadily, particularly in newly industrialized countries, with both incidence and prevalence increasing over the past decades. Canada currently reports one of the highest age-standardized incidence rates worldwide (26.8 per 1,00,000 persons), while Europe has reported the highest prevalence (UC: 505 per 1,00,000 in Norway; CD: 322 per 1,00,000 in Germany).^[1,2]^ More recent regional data suggest that incidence and prevalence across the Middle East and North Africa have continued to rise, reflecting this global trend.^[3,4]^

Vitamin D is a fat-soluble steroid hormone essential for skeletal health, primarily through regulation of calcium and phosphate metabolism. Beyond its classical role, vitamin D has been increasingly recognized for its immunomodulatory effects, possibly playing a role in autoimmune conditions.^[5]^ Vitamin D receptors (VDR) are expressed throughout the gastrointestinal tract and in immune cells, implicating vitamin D in the regulation of mucosal immunity and barrier integrity.^[6,7]^ Experimental models have shown that impaired vitamin D signaling can predispose and exaggerate autoimmune disorders, including IBD, and thus its targeted expression provided protective effects against developing colitis.^[8–12]^ Despite this, vitamin D deficiency is a widespread public health concern worldwide.

According to the Endocrine Society guidelines, deficiency is defined as serum 25-hydroxyvitamin D [25(OH)D] concentrations < 50 nmol/L, with insufficiency between 50 and 75 nmol/L, and sufficiency ≥ 75 nmol/L. Deficiency is particularly relevant in IBD, where it may arise due to a variety of reasons including reduced sunlight exposure, dietary insufficiency, VDR signaling, malabsorption, chronic inflammation, or medication use. Many studies in a meta-analysis have consistently shown that patients with IBD have higher odds of vitamin D deficiency compared to controls, though whether deficiency is a risk factor for IBD development or a consequence of disease remains unresolved.^[13]^

Overall, given the high prevalence of both IBD and vitamin D deficiency, this study aims to determine the prevalence of vitamin D deficiency among IBD patients and to assess its association with demographic and disease related extent and severity.

## 2. Materials and methods

### 2.1. Study design and ethical approval

This study utilized a cross-sectional comparative design, assessing an IBD case cohort from Mubarak Al-Kabeer Hospital, a tertiary healthcare IBD center in Kuwait, against a separate population-based control cohort. This approach compared serum vitamin D concentrations between patients with established IBD and a control group without IBD. Among the 2 major subgroups of IBD, CD and UC, patients were also examined for differences in vitamin D concentrations. This study adhered to the Strengthening the Reporting of Observational Studies in Epidemiology (STROBE) guidelines to ensure methodological rigor and transparency.

Ethical approval for this study was obtained from the Standing Committee for the Coordination of Health and Medical Research, Ministry of Health, Kuwait (Protocol No. 2501/2023). The requirement for informed consent was waived by the Standing Committee for Coordination of Health and Medical Research owing to the retrospective nature of the study and the use of de-identified data. The study was conducted in accordance with the principles of the Declaration of Helsinki (64th WMA General Assembly, Fortaleza, Brazil, October 2013) and the US Federal Policy for the Protection of Human Subjects.

### 2.2. Eligibility criteria

Data collection was conducted from September 21, 2025 until October 9, 2025. An initial screen identified 432 patients. Inclusion criteria were the following: patients with a confirmed diagnosis of IBD, including CD and UC, who attended Mubarak Al-Kabeer Hospital from September 1, 2020, until September 1, 2025; had vitamin D serum concentration measured at diagnosis, prior to initiation of any IBD-related medical therapy; no corticosteroid exposure within the preceding 12 months; age 18 to 65; and patients with moderate-to-severe ulcerative colitis defined as a clinical Mayo Score of >6, with endoscopic score of 2 to 3^[14]^; patients with moderate-to-severe Crohn disease defined as a Crohn Disease Activity Index [CDAI] > 220^[15]^; with Simple Endoscopic Score for Crohn Disease (SES-CD) ≥7.^[16]^

Exclusion criteria were: patients with random or no vitamin D concentration measurements, patients who have taken Vitamin D supplements within 1 year from date of measurement, patients with concomitant other chronic diseases, pregnancy, known diabetes (prior diagnosis and/or use of diabetes medication), inability to walk unaided, psychosis, terminal illness, and corticosteroid use within 12 months of vitamin D testing.

Controls (n = 410) from a population-based cross-sectional study were used in the current analysis and were subjected to additional eligibility criteria described below. Briefly, the Kuwait Wellbeing cross-sectional study enrolled participants between November 2012 and October 2017 aged 18 to 65 years, with no prior history of diabetes from all governorates of Kuwait.^[17]^ The cohort was screened with these set exclusionary criteria: no chronic diseases, including gastrointestinal conditions such as inflammatory bowel disease; patients who had taken Vitamin D supplementation within 1 year prior to serum 25-hydroxyvitamin D measurement.

### 2.3. Data collection

Demographic and clinical data, including age, sex, nationality, smoking status, and IBD type, were obtained from patient medical records. Serum 25-hydroxyvitamin D [25(OH)D] concentrations were measured using a chemiluminescent immunoassay (CLIA) on the DiaSorin LIAISON® analyzer (DiaSorin Inc.) employing the direct competitive immunoassay LIAISON® 25(OH) Vitamin D TOTAL Assay.^[18]^ Serum 25-hydroxyvitamin D [25(OH)D] was measured in nmol/L, and vitamin D concentrations were categorized according to Endocrine Society guidelines: severe deficiency (<25.0 nmol/L), deficiency (25.0–<50.0 nmol/L), insufficiency (50.0–<75.0 nmol/L), and sufficiency (≥75.0 nmol/L). Measurements between IBD patients and the control cohort were performed using the same assay platform and processed centrally in the same accredited laboratory to ensure methodological consistency between both groups. Diagnoses of inflammatory bowel disease (IBD), Crohn disease (CD), and ulcerative colitis (UC) were identified using the International Classification of Diseases, Tenth Revision (ICD-10). ICD-10 codes for Crohn disease under K50x. As for ulcerative colitis, under K51x, respectively.^[19]^

### 2.4. Sample size calculation

As this is a retrospective observational study, a formal sample size calculation was not applicable; instead, all consecutive eligible patients meeting the study criteria during the 2020 to 2025 enrollment period were included.

### 2.5. Statistical analysis

Continuous variables were reported as median and interquartile range (IQR), and categorical variables as frequencies and percentages. Statistical analyses were performed using SAS 9.4 (SAS Institute, Cary). The statistical significance level was set at α = 0.05 for all association analyses. Descriptive analyses were conducted to calculate the frequencies and proportions of categorical variables, and continuous variables were described by calculating their median and interquartile range (IQR). Univariable associations between categorical variables were evaluated using the chi-squared (χ^2^) test to assess differences in frequencies. Moreover, the Wilcoxon rank sum test (to compare 2 groups of a categorical variable) and the Kruskal–Wallis test (to compare 3 or more groups of a categorical variable) were applied to test for associations between an independent categorical variable and a continuous outcome variable.

Multivariable analyses were conducted to evaluate associations between 25(OH)D concentrations (exposure variable; modeled as a binary variable [Deficiency and In/Sufficiency (≥25.0) vs Severe deficiency (<25.0)] and as a continuous variable [per 10-unit increase]) and IBD status (dichotomous outcome variable) by applying binary logistic regression models to estimate adjusted odds ratios (aORs) and their 95% confidence intervals (CIs). To test whether the association between 25(OH)D concentrations and IBD status is modulated by sex, statistical interaction on a multiplicative scale was tested by including a product term (25(OH)D concentrations × sex) in the logistic regression models. Subsequently, associations between 25(OH)D concentrations and IBD status were stratified by sex. The Hosmer-Lemeshow goodness-of-fit test was used to evaluate model adequacy. Participants without available vitamin D measurements were excluded based on eligibility criteria. No imputation procedures were performed.

## 3. Outcome measures

The primary outcome of this study is to assess the prevalence of vitamin D deficiency (serum 25-hydroxyvitamin D < 50.0 nmol/L) in IBD patients compared to the general population. Secondary outcomes include IBD subtype comparison of vitamin D deficiency (CD vs UC), as well as the assessment of phenotypes of CD, or the extent of disease of UC, that may influence the degree of deficiency. Other outcomes include assessing cohort-specific demographics and their influence on possible vitamin D deficiency.

## 4. Results

A total of 432 patients were screened for eligibility. After applying inclusion and exclusion criteria, 184 patients with confirmed IBD were included in the final analysis.

### 4.1. Demographics

The demographic characteristics of the 594 study participants comprised 184 IBD patients and 410 controls. Among the IBD group, 122 (66.3%) had CD and 62 (33.7%) had UC. Males accounted for 53.8% of IBD patients and 56.8% of controls (*P* = .492). IBD patients were significantly younger than controls (median 31.0 vs 41.0 years, *P* < .001) and had a lower proportion of smokers (8.7% vs 39.0%, *P* < .001). The median serum 25-hydroxyvitamin D [25(OH)D] concentration was significantly lower in IBD patients than controls (18.0 vs 31.0 nmol/L, *P* < .001). Severe vitamin D deficiency (<25.0 nmol/L) was present in 78.8% of IBD patients compared with 36.3% of controls (*P* < .001; Table 1).

**Table 1:** Distribution of demographic characteristics and potential risk factors of inflammatory bowel disease (IBD) cases and controls.

	Cases (n = 184), n (%)	Controls (n = 410), n (%)	*P*-value
Sex			
Male	99 (53.8)	233 (56.8)	.492*
Female	85 (46.2)	177 (43.2)	
Age (completed yr)			
Median (IQR)	31.0 (26.0, 40.0)	41.0 (33.0, 49.0)	<.001†
<25	40 (21.7)	18 (4.4)	<.001*
25–34	73 (39.7)	99 (24.1)	
35–44	38 (20.7)	128 (31.2)	
45–54	16 (8.7)	118 (28.8)	
≥55	17 (9.2)	47 (11.5)	
Smoking status			
Smoker	16 (8.7)	160 (39.0)	<.001*
Nonsmoker	168 (91.3)	250 (61.0)	
Vitamin D (25(OH)D, nmol/L)			
Median (IQR)	18.0 (13.0, 23.0)	31.0 (20.0, 62.0)	<.001†
Severe deficiency (<25.0)	145 (78.8)	149 (36.3)	<.001*
Deficiency (≥25.0 to <50.0)	36 (19.6)	112 (27.3)	
Insufficiency (≥50.0 to <75.0)	2 (1.1)	100 (24.4)	
Sufficiency (≥75.0)	1 (0.5)	49 (12.0)	
IBD type			
Crohn disease (CD)	122 (66.3)	–	–
Ulcerative colitis (UC)	62 (33.7)	–	

25(OH)D = 25-hydroxyvitamin D, IBD = inflammatory bowel diseases, IQR = interquartile range (25th, 75th percentiles), CD = Crohn disease, UC = ulcerative colitis.

* Calculated using chi-squared (χ^2^) test.

† Calculated using the Wilcoxon rank sum test.

### 4.2. Primary outcome

When stratified by sex, median vitamin D concentrations were slightly lower in females (16.0 vs 19.0 nmol/L), with severe deficiency present in 83.5% of females and 74.8% of males among IBD patients. In contrast, the control group observed females scoring markedly higher median vitamin D concentrations than males (55.0 vs 27.0 nmol/L, *P* < .001; Table 2). Overall, IBD patients exhibited significantly higher odds of severe vitamin D deficiency compared with controls, where females demonstrated a stronger association (aOR 10.57, 95% CI: 5.33–20.96) than males (aOR 4.64, 95 % CI: 2.57–8.37; *P*_interaction_ = .021; Table S1, Supplemental Digital Content 1 and Fig. 1). Further, the continuous inverse association between vitamin D and IBD demonstrated that each 10 nmol/L increase in serum 25(OH)D was associated with approximately 50 % lower odds of IBD, consistent across both sexes (*P*_interaction_ = .436; Fig. 2; more information in Table S1, Supplemental Digital Content 1).

**Table 2:** Characteristics of inflammatory bowel disease (IBD) cases and controls stratified by sex.

	IBD cases			Controls			*P*-value [1 vs 3]	*P*-value [2 vs 4]
	Males [1] (n = 99), % (n)	Females [2] (n = 85), % (n)	*P*-value	Males [3] (n = 233), % (n)	Females [4] (n = 177), % (n)	*P*-value		
Age (completed yr)								
Median (IQR)	33.0 (28.0, 41.0)	29.0 (25.0, 39.0)	.093*	41.0 (32.0, 50.0)	42.0 (35.0, 48.0)	.873*	<.001*	<.001*
<25	20 (20.2)	20 (23.5)	.668†	7 (3.0)	11 (6.2)	.033†	<.001†	<.001†
25–34	36 (36.4)	37 (43.5)		67 (28.8)	32 (18.1)			
35–44	24 (24.2)	14 (16.5)		63 (27.0)	65 (36.7)			
45–54	9 (9.1)	7 (8.2)		70 (30.0)	48 (27.1)			
≥55	10 (10.1)	7 (8.2)		26 (11.2)	21 (11.9)			
Smoking status								
Smoker	13 (13.1)	3 (3.5)	.033†	139 (59.7)	21 (11.9)	<.001†	<.001†	.029†
Nonsmoker	86 (86.9)	82 (96.5)		94 (40.3)	156 (88.1)			
Vitamin D (25(OH)D, nmol/L)								
Median (IQR)	19.0 (13.0, 25.0)	16.0 (13.0, 22.0)	.103*	27.0 (20.0, 45.0)	55.0 (20.0, 70.0)	<.001*	<.001*	<.001*
Severe deficiency (<25.0)	74 (74.8)	71 (83.5)	.290†	95 (40.8)	54 (30.5)	<.001†	<.001†	<.001†
Deficiency (≥25.0 to <50.0)	22 (22.2)	14 (16.5)		87 (37.3)	25 (14.1)			
Insufficiency (≥50.0 to <75.0)	2 (2.0)	0 (0.0)		36 (15.5)	64 (36.2)			
Sufficiency (≥75.0)	1 (1.0)	0 (0.0)		15 (6.4)	34 (19.2)			
IBD type								
Crohn disease (CD)	69 (69.7)	53 (62.3)	.293†	–	–	–	–	–
Ulcerative colitis (UC)	30 (30.3)	32 (37.7)		–	–	–	–	–

25(OH)D = 25-hydroxyvitamin D, IBD = inflammatory bowel diseases, IQR = interquartile range (25th, 75th percentiles), CD = Crohn disease, UC = ulcerative colitis.

* Calculated using the Wilcoxon rank sum test.

† Calculated using chi-squared (χ^2^) test. If any cell count was <5 observations, Fisher exact test was used.

**Figure 1.**
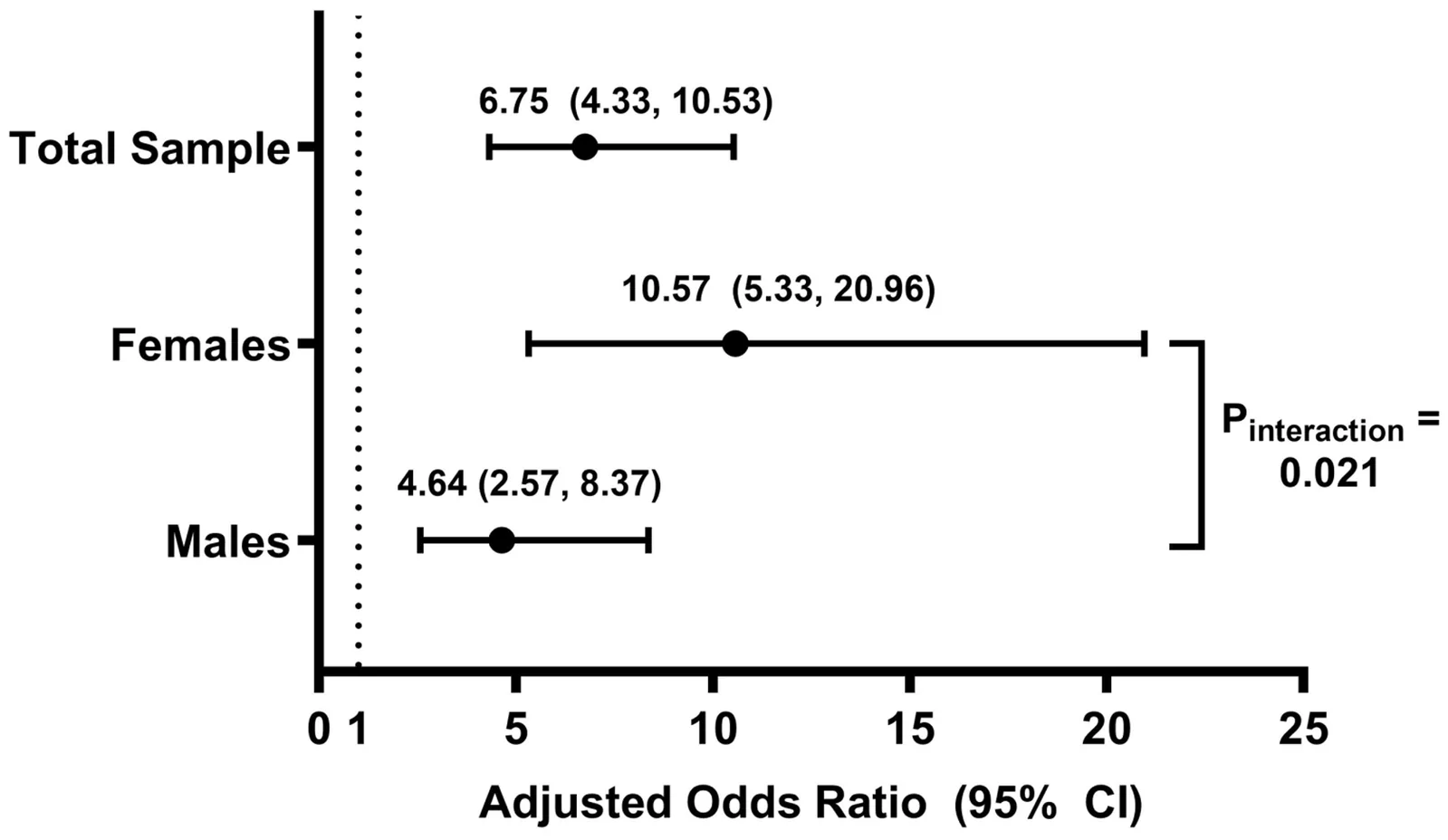
Association between severe 25-hydroxyvitamin D deficiency and inflammatory bowel disease (IBD) status. The forest plot displays the adjusted odds ratios (aOR) and 95% confidence intervals (CI) for IBD among individuals with severe vitamin D deficiency (<25.0 nmol/L) compared to a combined reference group of deficiency, insufficiency, and sufficiency (≥25.0 nmol/L). Estimates are presented for the total analytical sample and stratified by sex. The multivariable logistic regression model for the total sample was adjusted for sex, age, and smoking status. The sex-stratified models were adjusted for age and smoking status. Statistical interaction on a multiplicative scale was assessed by including a product term (“sex × 25-hydroxyvitamin D status”) in the total sample regression model. aOR = adjusted odds ratios, CI = confidence interval, 25(OH)D = 25-hydroxyvitamin D, IBD = inflammatory bowel diseases.

**Figure 2.**
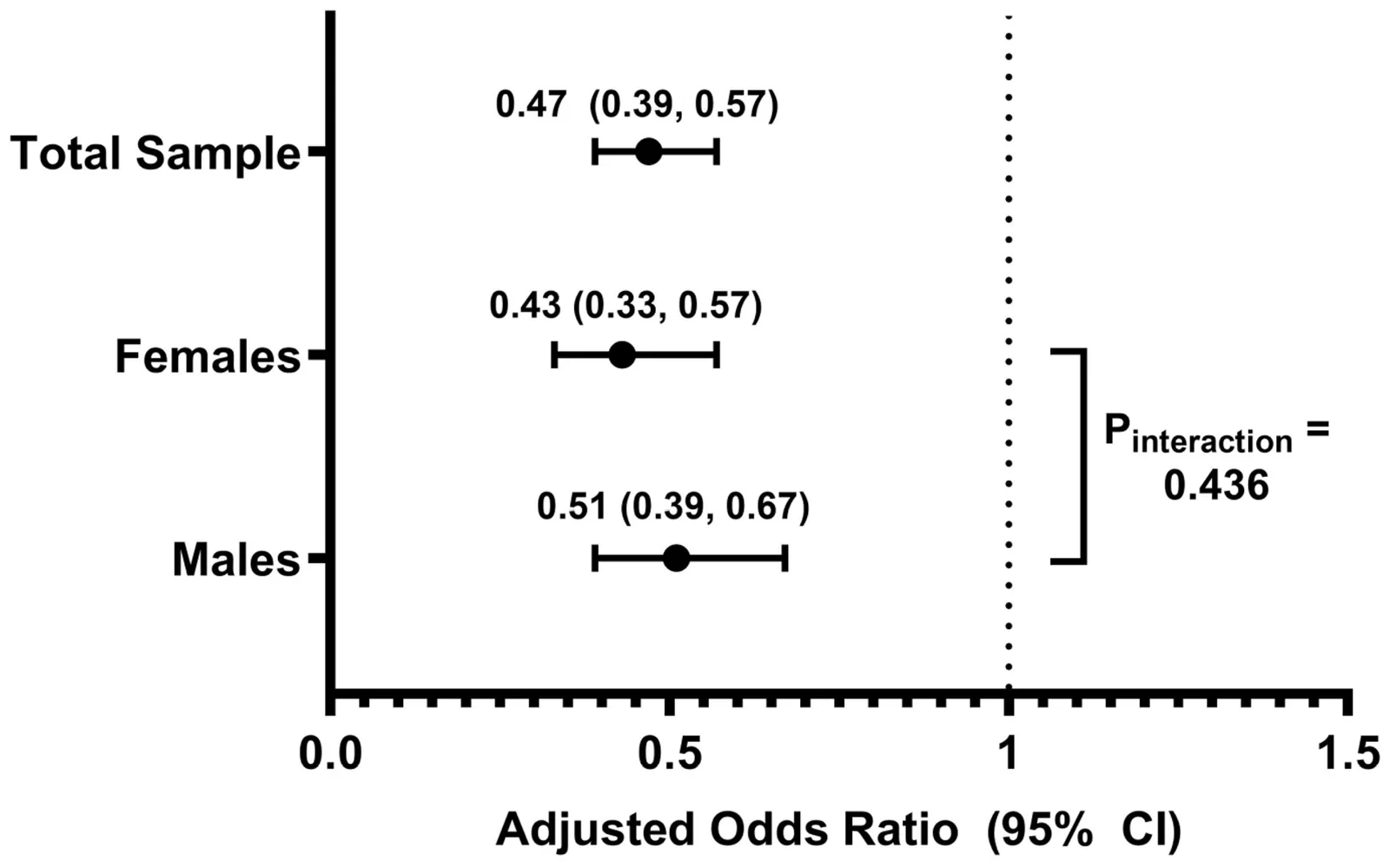
Association between continuous serum 25-hydroxyvitamin D concentrations and inflammatory bowel disease (IBD) status. The forest plot illustrates the adjusted odds ratios (aOR) and 95% confidence intervals (CI) for IBD per 10 nmol/L increase in serum 25(OH)D concentrations. Estimates are presented for the total analytical sample and stratified by sex. The multivariable logistic regression model for the total sample was adjusted for sex, age, and smoking status. The sex-stratified models were adjusted for age and smoking status. Statistical interaction on a multiplicative scale was evaluated by including a product term (“sex × 25-hydroxyvitamin D status”) in the total sample regression model. aOR = adjusted odds ratios, CI = confidence interval, 25(OH)D = 25-hydroxyvitamin D, IBD = inflammatory bowel diseases.

### 4.3. Secondary outcomes

When assessing IBD patients, vitamin D concentrations differed significantly, with a lower median in CD than UC (median 16.5 vs 22.0 nmol/L, *P* = .003; Table 3). Otherwise, no major differences can be appreciated across CD perianal disease status, CD behavior subtypes (B1–B3), CD location (L1–L3), or UC extent (E1–E3; Table S2, Supplemental Digital Content 2). Among females only, 25(OH)D differed by UC extent (*P* = .022), though the small subgroup sizes and lack of adjustment for multiple comparisons limit interpretation.

**Table 3:** Concentrations of 25-hydroxyvitamin D according to disease type in the total sample and stratified by sex.

	Crohn disease		Ulcerative colitis		*P*-value*
	n	25(OH)D (nmol/L), Median (IQR)	n	25(OH)D (nmol/L), Median (IQR)	
Total sample	122	16.5 (12.0, 22.0)	62	22.0 (15.0, 31.0)	.003
Males	69	18.0 (12.0, 22.0)	30	23.0 (16.0, 31.0)	.007
Females	53	16.0 (13.0, 22.0)	32	18.5 (12.5, 27.5)	.103
*P*-value*		.260		.121	

25(OH)D = 25-hydroxyvitamin D, IQR = interquartile range (25th, 75th percentiles).

* Calculated using the Wilcoxon rank sum test.

## 5. Discussion

This observational cross-sectional study investigated the relationship between vitamin D serum concentrations in IBD patients in Kuwait compared to those without. Here, we assess varying serum concentrations between different variables including sex, age, smoking status, and IBD type and subtypes. What we found was severe vitamin D deficiency was associated with markedly higher adjusted odds of IBD, with a stronger association in females (aOR 10.57, 95% CI: 5.33–20.96) than males (aOR 4.64, 95% CI: 2.57–8.37; P-interaction = 0.021). Moreover, when distinguishing among IBD patients, Crohn disease was associated with lower vitamin D concentrations compared to ulcerative colitis (Table 3), while no significant differences were observed across UC extent, CD location, behavior, or perianal disease status. A difference by UC extent was observed among females only, though the small subgroup sizes preclude firm conclusions. These findings underscore the consistently high burden of deficiency in IBD and are consistent with findings in separate studies.^[13,20-22]^

Sex-specific differences in vitamin D deficiency among IBD patients have been variably reported in the literature. Gubatan and Moss identified female sex as a predictor of deficiency,^[21]^ aligning with our observation that the association between severe vitamin D deficiency and IBD was more pronounced among women than men, and with broader literature outside of IBD.^[23]^ Conversely, other studies have reported the opposite pattern, with Prakash et al noting lower vitamin D concentrations among male IBD patients.^[24]^ A retrospective analysis by Pallav et al further suggested that sex may not be an independent predictor, reflecting mixed evidence across populations.^[25]^

Although females in our control group had significantly higher vitamin D concentrations than males, this pattern was reversed among IBD patients, implying that the presence of IBD modifies the relationship between sex and vitamin D. Females had a higher prevalence of deficiency and exhibited stronger odds of IBD under severe deficiency, whereas males appeared to manifest IBD even at relatively higher vitamin D concentrations, implying distinct biological sensitivity thresholds or immune susceptibilities. Notably, we observed a significant sex interaction when evaluating vitamin D status categorically (Pinteraction = 0.021), but this interaction lost significance in the continuous model (*P* interaction = .436; Table S1, Supplemental Digital Content 1). This divergence is compatible with a nonlinear threshold effect rather than a uniform linear dose-response. It suggests that sex-specific disparities in IBD do not manifest gradually across the entire spectrum of vitamin D decline, but rather emerge sharply only when a critical state of severe depletion (<25 nmol/L) is reached. While this could be plainly attributed to factors such as greater supplementation use and preventative care among women,^[26]^ these differences could also be attributed to different hormonal regulation. For instance, estrogen has been shown to up-regulate VDR gene expression and enhance vitamin D responsiveness^[27-29]^ as well as reduce the disease process.^[30]^ In autoimmune and inflammatory models, vitamin D’s immunomodulatory effects (e.g., suppressing T cell activation or pro-inflammatory cytokines) have been more potent in females than in males.^[27,28,31]^ This may explain the higher concentrations observed in controls but appear insufficient once disease-related mechanisms such as malabsorption and chronic inflammation cause deficiency. Overall, our findings highlight that IBD not only increases the overall burden of vitamin D deficiency but also disproportionately affects women, reversing trends observed in the general population.

The relationship between IBD subtype and vitamin D deficiency remains largely debated in the literature. A meta-analysis by Li et al reported that both CD and UC patients exhibit lower vitamin D concentrations compared to controls but found no significant difference between the 2 subtypes.^[20]^ Similar observations were seen in a pediatric IBD cohort.^[32]^ Contrarily, other studies do note more frequent associations between vitamin D deficiency in CD compared to UC,^[25,33,34]^ a finding that mirrors our own; however, some indicate that subtype may not be independently associated with deficiency when adjusting for confounders.^[25]^

Beyond subtypes, we also explored whether disease extent or phenotype modifies vitamin D concentrations. Here, our findings were inconclusive, with no clear association of disease extent. A Polish study in 2022 also echoed similar results.^[35]^ However, in a Chinese cohort of 862 CD patients, penetrating behavior (Montreal B3) was independently associated with severe vitamin D deficiency. This suggests more aggressive disease behavior contributes to nutritional compromise.^[36]^ Collectively, phenotype and extent may influence deficiency in certain populations, though findings are still inconsistent. As such, this warrants further research in a larger well-defined cohort to address vitamin D deficiency as well as its varying degree of nutritional impact.

In our study, serum vitamin D concentrations were measured at the time of diagnosis, prior to the initiation of medical therapy, and patients with recent corticosteroid exposure were excluded. These design features were implemented to minimize medication-related effects on vitamin D levels. Therapeutic modalities, such as systemic corticosteroids, have been associated with impaired calcium absorption, altered vitamin D metabolism, and reduced bone mineral density, particularly with prolonged exposure.^[37,38]^ In addition, disease activity, particularly clinical severity, may influence vitamin D status through reduced physical activity, dietary changes, and impaired intestinal absorption.^[5]^ As such, treatment intensity often can reflect disease severity, which may parallel the lower vitamin D concentration observed. Notably, some studies have suggested that, in patients who are starting anti-tumor necrosis factor (anti-TNF) therapy, vitamin D deficiency may be associated with early cessation of treatment or possibly a reduction in overall response. Whether this reflects disease severity or a direct influential role of vitamin D remains unclear.^[39]^

The main strengths of this study lie in its relatively large cohort size and the use of a population-based comparison group. Serum 25(OH)D was measured on the same assay platform and processed centrally in 1 accredited laboratory for both cases and controls, ensuring methodological consistency between groups. The analysis further incorporated multiple demographic and clinical variables such as IBD type and subtype, providing a more comprehensive assessment of associated factors. However, several limitations should be acknowledged. First, the retrospective design limits causal inference and may be subject to incomplete or missing data. Second, potential confounders such as dietary intake, unrecorded vitamin D supplementation obtained outside the healthcare system (e.g., over-the-counter use), and seasonal variation in sunlight exposure were not captured. Furthermore, the control cohort was sampled over an earlier multi-year period (November 2012 to October 2017), whereas the IBD cohort was diagnosed between 2020 and 2025. While this temporal gap and potential variations in the specific seasons of blood collection could theoretically introduce bias in the observed 25(OH)D concentrations, a prior analysis of the Kuwait Wellbeing cohort demonstrated no significant differences in 25(OH)D levels across the different seasons of blood collection.^[19]^ It should also be noted that the highly limited number of IBD patients in the insufficiency (n = 2) and sufficiency (n = 1) categories reduces the precision of descriptive interpretations for these specific categories, even though robust testing was applied. To overcome this limitation and ensure statistical stability in our multivariable modeling, these upper categories were collapsed with the deficiency group (≥25.0 nmol/L) to serve as a unified reference category against severe deficiency (<25.0 nmol/L). In addition, cases and controls differed at baseline with respect to age (median 31.0 vs 41.0 years) and smoking status (8.7% vs 39.0% smokers). Although both variables were adjusted for in all multivariable models, residual confounding cannot be excluded from imperfectly measured or unmeasured aspects of these factors. Lastly, as a single-center study, the findings may not be fully generalizable to the broader Kuwaiti or regional IBD population.

## 6. Conclusion

Vitamin D deficiency is highly prevalent among patients with inflammatory bowel disease, particularly in those with Crohn disease and in females, underscoring both systemic disease-related associations and sex-specific vulnerability. Given the potential implications for disease outcomes, routine screening and appropriate supplementation should be considered as part of comprehensive IBD management. Larger, prospective, multicenter studies are warranted to further elucidate the associated factors and clinical implications of vitamin D deficiency in this population.

## Author contributions

**Data curation:** Ali H. Ziyab.

**Formal analysis:** Ali H. Ziyab.

**Investigation:** Ali H. Ziyab.

**Methodology:** Ali H. Ziyab.

**Software:** Ali H. Ziyab.

**Supervision:** Mohammad Shehab.

**Writing – original draft:** Mohammad Shehab, Abdulwahab AlSayegh, Ali H. Ziyab.

**Writing – review & editing:** Mohammad Shehab, Abdulwahab AlSayegh.




