# Supplementary material for: Relationship between vitamin D serum concentration and inflammatory bowel disease demographics: A cross-sectional study
Source: Medicine (Baltimore). 2026 Oct 16;105(42):e51257. doi: 10.1097/MD.0000000000051257 (PMC13681150; doi:10.1097/MD.0000000000051257)
Supplement: Supplementary file 1 [file medi-105-e51257-s001.docx]

**Supplementary Table 1.** Associations between 25-hydroxyvitamin D status and IBD status stratified by sex

|  | **Total sample** |  |  | **Males** | |  | **Females** | |  |
| --- | --- | --- | --- | --- | --- | --- | --- | --- | --- |
| **25(OH)D (nmol/L)** | **aOR (95% CI)** | ***P*-value** |  | **aOR (95% CI)** | ***P*-value** |  | **aOR (95% CI)** | ***P*-value** | **P_interaction_^‡^** |
| **Model 1** |  |  |  |  |  |  |  |  |  |
| Deficiency and In/Sufficiency (≥25.0) | 1.00 (Reference) |  |  | 1.00 (Reference) | – |  | 1.00 (Reference) | – | 0.021 |
| Severe deficiency (<25.0) | 6.75 (4.33, 10.53) | <0.001 |  | 4.64 (2.57, 8.37) | <0.001 |  | 10.57 (5.33, 20.96) | <0.001 |  |
| **Model 2** |  |  |  |  |  |  |  |  |  |
| Per 10-unit increase in 25(OH)D | 0.47 (0.39, 0.57) | <0.001 |  | 0.51 (0.39, 0.67) | <0.001 |  | 0.43 (0.33, 0.57) | <0.001 | 0.436 |

25(OH)D: 25-hydroxyvitamin D; aOR: adjusted odds ratio; CI: confidence interval.

^†^ Adjusted for age and smoking status.

^‡^ In the total analytical sample that included data of males and females, the interaction was assessed by including the following product term in the regression model: ‘sex × 25-hydroxyvitamin D status.
