# Supplementary material for: Relationship between vitamin D serum concentration and inflammatory bowel disease demographics: A cross-sectional study
Source: Medicine (Baltimore). 2026 Oct 16;105(42):e51257. doi: 10.1097/MD.0000000000051257 (PMC13681150; doi:10.1097/MD.0000000000051257)
Supplement: Supplementary file 2 [file medi-105-e51257-s002.docx]

**Supplementary Table 2.** Levels of 25-hydroxyvitamin D according to subtypes of Crohn’s disease (CD) and Ulcerative colitis (UC)

|  | **Total Sample** | |  | **Males** | |  | **Females** | |
| --- | --- | --- | --- | --- | --- | --- | --- | --- |
| **CD location** | **n** | **25(OH)D (nmol/L),**  **Median (IQR)** |  | **n** | **25(OH)D (nmol/L),**  **Median (IQR)** |  | **n** | **25(OH)D (nmol/L),**  **Median (IQR)** |
| L1 | 56 | 17.0 (12.5, 22.0) |  | 29 | 17.0 (11.0, 22.0) |  | 27 | 15.0 (13.0, 22.0) |
| L2 | 10 | 16.5 (12.0, 21.0) |  | 7 | 17.0 (12.0, 21.0) |  | 3 | 16.0 (11.0, 25.0) |
| L3 | 47 | 18.0 (13.0, 23.0) |  | 28 | 18.0 (12.5, 24.5) |  | 19 | 16.0 (14.0, 22.0) |
| ***P-value*^‡^** |  | *0.735* |  |  | *0.721* |  |  | *0.871* |
| **CD behavior** |  |  |  |  |  |  |  |  |
| B1 | 55 | 18.0 (12.0, 22.0) |  | 29 | 19.0 (12.0, 22.0) |  | 26 | 17.5 (13.0, 22.0) |
| B2 | 28 | 15.5 (12.0, 22.0) |  | 13 | 15.0 (11.0, 22.0) |  | 15 | 15.0 (11.0, 22.0) |
| B3 | 33 | 17.0 (13.0, 22.0) |  | 24 | 17.5 (12.5, 24.0) |  | 9 | 17.5 (12.5, 24.0) |
| ***P-value*^‡^** |  | *0.837* |  |  | *0.899* |  |  | *0.842* |
| **CD Perianal** |  |  |  |  |  |  |  |  |
| Yes | 18 | 15.0 (11.0, 22.0) |  | 16 | 16.5 (11.0, 25.0) |  | 2 | 15.0 (15.0, 15.0) |
| No | 55 | 16.0 (12.0, 22.0) |  | 25 | 18.0 (13.0, 23.0) |  | 30 | 15.5 (11.0, 19.0) |
| ***P-value*^‡^** |  | *0.833* |  |  | *0.883* |  |  | *0.876* |
| **UC inflammation** |  |  |  |  |  |  |  |  |
| E1 | 20 | 23.0 (13.5, 32.5) |  | 12 | 22.5 (11.5, 31.5) |  | 8 | 27.5 (17.5, 33.0) |
| E2 | 12 | 23.0 (17.5, 28.0) |  | 6 | 22.5 (16.0, 27.0) |  | 6 | 23.0 (19.0, 29.0) |
| E3 | 28 | 17.0 (12.5, 26.0) |  | 11 | 25.0 (17.0, 33.0) |  | 17 | 15.0 (10.0, 18.0) |
| ***P-value*^‡^** |  | *0.325* |  |  | *0.498* |  |  | ***0.022*** |

25(OH)D: 25-hydroxyvitamin D; IQR: interquartile range (25^th^, 75^th^ percentiles).

^‡^ Calculated using the Kruskal-Wallis test.
